# FUNCTIONAL LIMITATIONS, LIVING ARRANGEMENT, DAILY SOCIAL CONTACTS, AND LONELINESS IN LATE LIFE

**DOI:** 10.1093/geroni/igad104.0262

**Published:** 2023-12-21

**Authors:** Shiyang Zhang

**Affiliations:** The University of Texas at Austin, Austin, Texas, United States

## Abstract

Older adults with functional limitations are at a greater risk of social isolation and loneliness, largely due to difficulties in mobility and compromised social contacts. Lack of social contact throughout the day may contribute to loneliness among older adults with functional limitations, and these associations may differ by living arrangements. This study examined associations between functional limitations, living alone, daily social contacts, and loneliness throughout the day. Community-dwelling older adults aged 65 + (N = 310, Mage = 73.96) from the Daily Experiences and Well-being Study completed a measure of functional limitations in a baseline interview. Then, in ecological momentary assessments (EMA), they reported social contacts and loneliness every 3 hours for 5 to 6 days. Multilevel models revealed a significant interaction between functional limitations and social contacts on loneliness, such that people with higher levels of functional limitations felt less lonely if they had more social contacts in the prior 3 hours, but not for those with lower levels of functional limitations. The three-way interaction between functional limitations, living alone, and loneliness revealed that more social contacts were associated with reduced loneliness only among individuals with functional limitations and who lived with others. Individuals with functional limitations who lived alone had higher levels of loneliness than those with functional limitations who lived with others. The findings emphasize the vulnerability in social isolation of older adults with functional limitations, especially those who live alone. Yet this vulnerable group may also benefit more from adequate daily social contacts.

